# The Relationship Between Personality Types and Attitudes Toward Psychiatry Among Medical and Psychology Students in the United Arab Emirates: A Cross-Sectional Study

**DOI:** 10.1192/bjo.2025.10155

**Published:** 2025-06-20

**Authors:** Syed Fahad Javaid, Jigna Stott, Gabriel Andrade, Nusrat Khan

**Affiliations:** 1 Department of Psychiatry, College of Medicine and Health Sciences, United Arab Emirates University, Al Ain, UAE; 2 College of Medicine, Ajman University, Ajman, United Arab Emirates, Ajman, UAE; 3 Mohammed Bin Rashid University of Medicine and Health Sciences, Dubai, UAE

## Abstract

**Aims:** While personality traits are known to influence values, beliefs, and professional preferences, limited research has explored their impact on attitudes toward psychiatry, particularly in Middle Eastern contexts and among non-medical students. Personality traits also influence perceptions of mental illness among individuals, helping to understand the basis of societal mental health stigma. Understanding the relationship between personality traits and attitudes toward psychiatry is crucial for developing educational strategies that support positive perceptions, ultimately enhancing healthcare outcomes for individuals living with mental illnesses.

**Methods:** This study employed a cross-sectional design to evaluate how personality dimensions correlate with perceptions of psychiatry in a sample of 503 students, including 377 medical and 126 psychology students from three major universities in the United Arab Emirates. Attitudes toward psychiatry were assessed using the Attitudes Towards Psychiatry (ATP-30) scale, and personality traits were evaluated using the Big Five Inventory. Spearman’s rank correlation was employed to analyse the relationships between ATP scores and personality dimensions: extraversion, agreeableness, conscientiousness, emotional stability, and openness to experience.

**Results:** Significant positive correlations were found between ATP-30 scores and four personality traits: extraversion (r=0.11, p=0.01), agreeableness (r=0.22, p<0.001), conscientiousness (r=0.10, p=0.03), and openness (r=0.28, p<0.001). Emotional stability did not exhibit a significant correlation (r=−0.03, p=0.58). Medical students demonstrated similar patterns, with openness (r=0.26, p<0.001) and agreeableness (r=0.20, p<0.001) being the strongest predictors. Among psychology students, only agreeableness (r=0.21, p=0.02) and openness (r=0.30, p<0.001) showed significant associations.

**Conclusion:** These findings underscore the influence of personality traits – particularly agreeableness and openness – on attitudes toward psychiatry. They highlight the need for tailored educational approaches to promote positive perceptions of psychiatry, with implications for improving both training outcomes and therapeutic relationships.

